# Hemophilia in the Era of Advanced Therapies: Structural Monitoring, the Role of Musculoskeletal Ultrasound, and a Proposed Multidisciplinary Care Model—A Structured Narrative Review

**DOI:** 10.3390/diagnostics16111683

**Published:** 2026-05-29

**Authors:** Felipe Querol-Giner, Magdalena Querol-Giner, Sofía Pérez-Alenda, Felipe Querol-Fuentes

**Affiliations:** 1 PTinMotion Research Group, Department of Physiotherapy, University of Valencia, 46010 Valencia, Spain; sofia.perez-alenda@uv.es; 2 Department of Rehabilitation, Hospital Clínico Universitario de Valencia, 46010 Valencia, Spain; 3 Faculty of Physiotherapy, University of Valencia, 46010 Valencia, Spain; felipe.querol@uv.es

**Keywords:** advanced therapies, hemophilia, hemophilic arthropathy, hemostatic control, musculoskeletal ultrasound, point-of-care ultrasound, rehabilitation, structural monitoring

## Abstract

**Background:** Advances in hemophilia treatment, including extended half-life factor concentrates, non-replacement therapies, and gene therapy, have substantially reduced bleeding frequency and improved life expectancy. However, persistent musculoskeletal damage, subclinical bleeding, and residual arthropathy remain important clinical challenges despite improved hematologic control. **Objective:** We aimed to analyze recent therapeutic advances in hemophilia, examine persistent musculoskeletal complications, and propose a multidisciplinary care model based on structural monitoring, highlighting the role of musculoskeletal ultrasound. **Methods:** A structured narrative review with a reproducible search strategy was conducted following PRISMA 2020-informed methodological principles. PubMed/MEDLINE and Scopus were searched for clinically relevant studies published between 2018 and 2026 focusing on advanced hemophilia therapies, musculoskeletal complications, and structural monitoring. A total of 478 records were identified, and 13 studies were included after screening and selection using the Rayyan platform. **Results:** Modern therapies markedly reduce clinically evident hemarthroses, but structural joint alterations and subclinical disease activity may persist, particularly in patients with pre-existing arthropathy. Imaging-based studies identified persistent synovial and osteochondral alterations despite effective hematologic control. Magnetic resonance imaging remains the reference standard for structural assessment, although its routine use may be limited by accessibility and cost. Musculoskeletal ultrasound emerges as an accessible and reproducible tool for dynamic joint evaluation and early detection of structural alterations, supporting longitudinal monitoring and individualized rehabilitation strategies. **Conclusions:** In the era of advanced therapies, comprehensive hemophilia management requires not only effective hematologic control but also structured musculoskeletal follow-up. The integration of musculoskeletal ultrasound into multidisciplinary care models may favor earlier detection of joint alterations and more individualized rehabilitation strategies.

## 1. Introduction

Hemophilia is an inherited bleeding disorder characterized by a congenital deficiency of clotting factors VIII (hemophilia A) or IX (hemophilia B). The disease is classified according to plasma factor activity as severe (<1%), moderate (1–5%), or mild (>5%). Recurrent bleeding episodes into joints—particularly the knees, ankles, and elbows—represent the main cause of functional impairment. Repeated hemarthroses trigger inflammatory processes, chronic synovitis, and progressive cartilage degeneration, leading to hemophilic arthropathy [1,2], whose structural progression plays a key role in long-term functional outcomes.

For much of the twentieth century, hemophilia was associated with high musculoskeletal morbidity and significantly reduced life expectancy. The development of factor concentrates and, more recently, recombinant products has led to a major therapeutic shift, particularly with the implementation of regular prophylaxis. However, access to these treatments remains highly unequal: according to the World Federation of Hemophilia (WFH) global survey [3], approximately 80% of people with hemophilia worldwide lack continuous prophylaxis or specialized follow-up.

New hematologic agents—extended half-life (EHL) products, bispecific antibodies such as emicizumab, and gene therapies—have transformed the clinical and functional landscape, markedly reducing bleeding rates and enabling more active and independent lifestyles [4]. Recent evidence has shown that modern prophylactic strategies are associated with improvements in health-related quality of life, particularly regarding physical activity, psychosocial well-being, and participation in daily activities among people with hemophilia [5]. However, improved hematologic control does not eliminate pre-existing musculoskeletal damage or the risk of subclinical joint deterioration [6,7]. The profile of patients with hemophilia is evolving: there is a lower frequency of clinically evident hemarthroses but a higher prevalence of age-related comorbidities, chronic musculoskeletal sequelae, and residual arthropathy [8]. In this context, hematologic control can no longer be considered synonymous with preservation of joint structural integrity, highlighting the need for more sensitive and specific monitoring strategies, particularly in patients with minimal symptoms but potential underlying structural progression. Despite these advances, there remains a lack of standardized approaches to structural monitoring capable of accurately correlating hematologic control with joint outcomes. Although magnetic resonance imaging (MRI) is considered the reference standard for structural joint assessment, its cost and limited accessibility restrict its use in routine follow-up.

In this new scenario, physiotherapy and rehabilitation have acquired a strategic role. Their focus is no longer limited to post-bleeding recovery but extends to prevention, functional maintenance, and patient education in safe exercise, taking advantage of improved hematologic control to optimize quality of life. However, optimizing rehabilitation strategies requires objective tools capable of detecting early structural changes, even in the absence of overt clinical symptoms [9].

The aim of this review is to analyze recent therapeutic advances, describe persistent musculoskeletal challenges, and examine the need for systematic structural monitoring, highlighting the strategic role of musculoskeletal ultrasound (MSK-US) as a monitoring tool in clinical practice within a multidisciplinary care model focused on prevention and functional preservation.

## 2. Materials and Methods

A structured narrative review was conducted, following PRISMA 2020-informed methodological principles [10], to identify clinically relevant literature on hemophilia, musculoskeletal complications, and structural monitoring. A PRISMA 2020 checklist indicating the applicable items is available in the Supplementary Materials. Given the clinically integrative nature of this structured narrative review, the research objective was not formulated according to a strict PICO (Population, Intervention, Comparison, and Outcome) framework.

### 2.1. Search Strategy

The literature search was performed in the PubMed/MEDLINE and Scopus databases, including studies published between January 2018 and April 2026. To prioritize clinically relevant literature and reduce the retrieval of excessively broad or non-specific records, search terms were restricted to the title and abstract fields (Title/Abstract in PubMed; TITLE-ABS in Scopus). The search strategy was based on a primary equation focused on advanced hematologic therapies and their musculoskeletal impact, applied consistently across both databases to ensure coherence and reproducibility in the identification of studies. The complete search strategy is reported to ensure reproducibility.

The search equations used in the different databases were as follows:

- PubMed (main block):

(hemophilia [Title/Abstract] OR haemophilia [Title/Abstract]) AND (emicizumab [Title/Abstract] OR “extended half-life” [Title/Abstract] OR “gene therapy” [Title/Abstract] OR fitusiran [Title/Abstract] OR concizumab [Title/Abstract]) AND (musculoskeletal [Title/Abstract] OR arthropathy [Title/Abstract] OR joint [Title/Abstract])

- Scopus (main block):

TITLE-ABS (hemophilia OR haemophilia) AND TITLE-ABS (emicizumab OR “extended half-life” OR “gene therapy” OR fitusiran OR concizumab) AND TITLE-ABS (musculoskeletal OR arthropathy OR joint)

The following criteria were applied:

- Studies in humans (applied through filters in PubMed and as a selection criterion in Scopus);
- Language: English and Spanish.

No restrictions were applied regarding study design in the search strategy in order to maximize sensitivity. Final selection was performed during the screening process, prioritizing original articles, reviews, clinical guidelines, and consensus documents with direct clinical relevance to the study objective.

### 2.2. Reference Management and Study Selection

All identified references were exported to the Rayyan platform (Qatar Computing Research Institute, Doha, Qatar) for the management of the selection process.

The following steps were performed:

1. Duplicate removal, using the platform’s automated tools, based on combined criteria including title, authors, year of publication, and digital object identifier (DOI), as well as overall similarity between records. This process was complemented by manual review to resolve potential discrepancies and ensure accurate data cleaning.
2. Title and abstract screening.
3. Full-text assessment of potentially eligible articles.

The selection process was conducted in a structured and reproducible manner, prioritizing thematic consistency with the objectives of the review.

Data extraction was performed focusing on study characteristics, patient population, type of therapy, musculoskeletal outcomes, and imaging findings, allowing a thematic synthesis of the evidence.

### 2.3. Inclusion and Exclusion Criteria

Inclusion criteria:

- Studies with direct clinical applicability in hemophilia.
- Studies addressing:
  - Musculoskeletal complications;
  - Hemophilic arthropathy;
  - Subclinical bleeding;
  - MSK-US;
  - Rehabilitation strategies.
- Clinical guidelines and international consensus documents.
- Relevant reviews and clinical studies.

Review articles were considered eligible when they provided clinically relevant integrative information regarding musculoskeletal complications, structural monitoring strategies, rehabilitation approaches, or multidisciplinary care models in hemophilia.

Exclusion criteria:

- Studies not directly related to the objective of the review;
- Basic or experimental research without clinical applicability;
- Isolated case reports without generalizable value;
- Letters to the editor or opinion papers without relevant clinical content;
- Duplicate or redundant articles.

### 2.4. Final Selection Strategy

The final selection of studies was aimed at constructing a coherent, up-to-date, and clinically applicable body of evidence, with particular emphasis on musculoskeletal involvement and its structural assessment.

During full-text evaluation, additional selection criteria were applied based on the clinical relevance and functional content of the studies. In particular, priority was given to studies that included:

- Assessment of joint involvement in patients with hemophilia;
- Use of clinical assessment tools and/or imaging techniques (such as the Hemophilia Joint Health Score [HJHS], MSK-US, or MRI);
- Clinical applicability of findings in patient follow-up and management.

Studies that met the initial search criteria but did not provide specific information on musculoskeletal assessment, did not include structural or functional evaluation tools, or had an overly general approach without direct clinical applicability to the study objective were excluded. This selective approach was intentionally adopted to maintain a focused review specifically centered on clinically applicable musculoskeletal outcomes, structural monitoring, and rehabilitation-related implications in hemophilia.

### 2.5. Methodological Considerations

Given the narrative and clinically oriented nature of the review, no formal risk of bias assessment tools (such as Risk of Bias 2 [ROB 2] or STROBE) were applied, with priority given instead to the clinical relevance and applicability of the included studies.

However, specific measures were implemented to minimize potential sources of bias during the selection process, particularly those related to reviewer fatigue and sequential decision-making.

Full-text screening was conducted across multiple sessions over time, avoiding prolonged continuous evaluation of large volumes of articles. A limited number of studies was assessed per session, which helped maintain consistency in the selection criteria and reduce the risk of inconsistent decisions.

In addition, articles initially classified as “uncertain” (Rayyan “maybe” category) were deliberately reassessed in separate sessions, allowing for a more reflective second evaluation and minimizing the risk of premature exclusion bias.

The selection process followed a progressive refinement approach, maintaining consistent clinical criteria across all screening phases in order to ensure methodological coherence and reproducibility.

Due to the methodological heterogeneity of the included studies, two different coding approaches were applied. Structured assessment tools and outcome measures (including HJHS, MRI, MSK-US, ABR, and specific functional measurements) were coded as present only when they were explicitly applied or evaluated as part of the study methodology or outcomes. In contrast, broader musculoskeletal concepts such as synovitis, subclinical bleeding, and hemophilic arthropathy were additionally coded when they represented a central analytical focus of the article, including in clinically oriented narrative reviews. This approach was considered particularly relevant given that the objectives of the present structured narrative review were not limited to identifying the use of structured musculoskeletal assessment tools, but also to contextualizing clinically relevant musculoskeletal aspects related to synovitis, subclinical bleeding, structural joint involvement, and multidisciplinary management in the era of advanced hemophilia therapies.

Additionally, a limited number of complementary references were consulted exclusively to contextualize specific aspects of the discussion (such as changes in life expectancy or the impact of emerging therapies), although these references were not included in the structured study selection process nor considered part of the primary evidence synthesis or the core findings derived from the selected studies.

This review follows PRISMA 2020 principles to ensure transparency in study identification, screening, and selection, aligned with the scope and objectives of the present structured narrative review, allowing it to be represented through a flow diagram, although the study was not designed as a formal systematic review.

### 2.6. Flow of Study Selection

**Figure 1:**
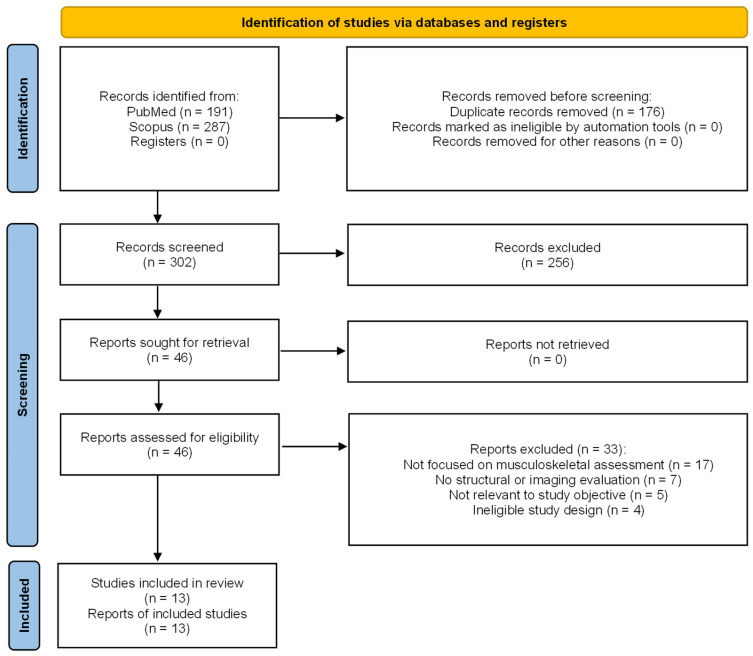
Flow diagram of the identification, screening, and selection process based on the PRISMA 2020 guidelines.

The study identification and selection process is summarized in Figure 1 (PRISMA flow diagram). A total of 478 records were identified, of which 13 studies were ultimately included in the review.

## 3. Results

### 3.1. Characteristics of Included Studies

A total of 13 studies were included in this review, following the predefined selection process, comprising prospective observational studies, cross-sectional analyses, and review articles. The included studies evaluated patients with hemophilia A across different age groups, including pediatric and adult populations, with varying disease severity and treatment regimens.

Most studies focused on the musculoskeletal impact of modern therapeutic strategies, including EHL factor concentrates and non-replacement therapies such as emicizumab, as well as on joint assessment methods, particularly using clinical scoring systems and imaging techniques such as MRI and MSK-US [11,12,13,14,15].

The main characteristics of the included studies are summarized in Table 1, which details study design, population, variables assessed, and key clinical findings for each study.

MSK-US was reported in 46.2% of studies, HJHS in 38.5%, MRI in 15.4%, and ABR or bleeding-related outcomes in 38.5% of studies [11,12,13,14,15]. Specific independent measurements of range of motion, pain, or muscle function were reported in 7.7% of studies.

In contrast, broader musculoskeletal concepts such as synovitis, subclinical bleeding, and hemophilic arthropathy appeared in 76.9%, 46.2%, and 76.9% of studies, respectively, reflecting their recurrent role as central clinical, thematic, or interpretative focuses within the included literature, even when they were not directly evaluated as structured methodological outcomes, including clinically oriented narrative reviews in which these concepts constituted a central analytical focus.

**Figure 2:**
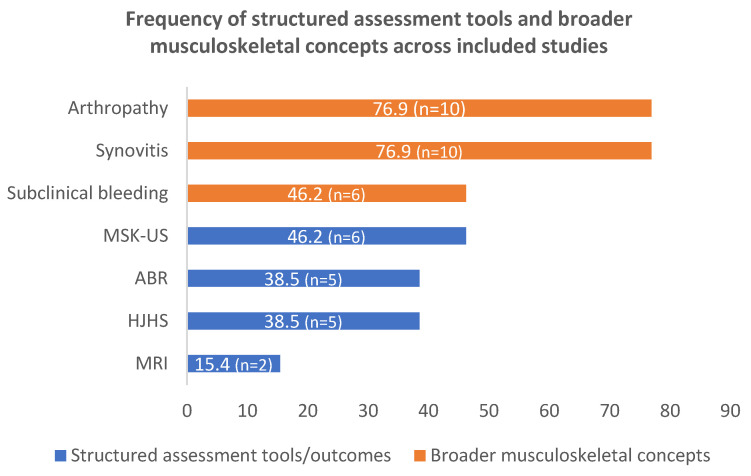
Frequency of explicitly measured/evaluated structured assessment tools and outcomes (blue bars) and broader musculoskeletal concepts included as thematic or interpretative focuses without specific structured measurement (orange bars) across included studies. ABR: annualized bleeding rate; HJHS: Hemophilia Joint Health Score; MRI: magnetic resonance imaging; MSK-US: musculoskeletal ultrasound.

**Figure 3:**
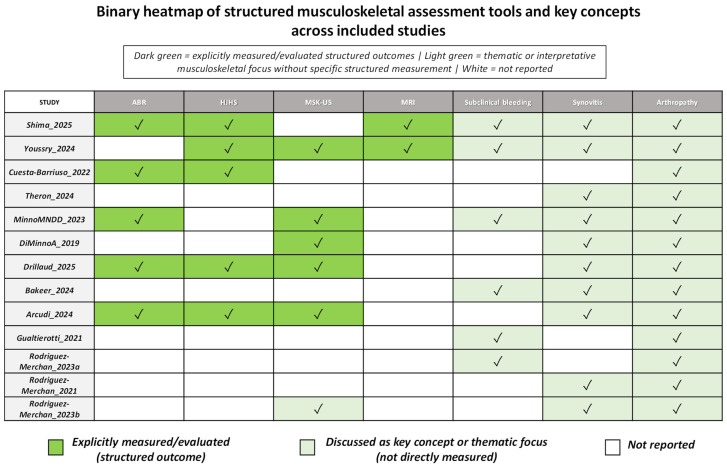
Binary heatmap of structured musculoskeletal assessment tools and outcomes across included studies. ABR: annualized bleeding rate; HJHS: Hemophilia Joint Health Score; MRI: magnetic resonance imaging; MSK-US: musculoskeletal ultrasound.

Figure 2 summarizes the frequency of structured musculoskeletal assessment tools and outcome measures explicitly applied across the included studies. Figure 3 presents a binary heatmap integrating both structured assessment tools and broader musculoskeletal concepts, visually differentiating studies providing direct methodological assessment from those contributing primarily thematic or interpretative musculoskeletal perspectives.

Collectively, the original clinical studies with directly reportable patient populations included in this review evaluated approximately 250 patients with hemophilia across pediatric and adult populations, reflecting a broad spectrum of disease severity, therapeutic strategies, and musculoskeletal involvement (Table 2). Review articles, narrative reviews, and conceptual papers were not included in this cumulative estimation because they did not provide independent quantifiable patient cohorts.

### 3.2. Impact of Modern Therapies on Bleeding Events

Across the included studies, modern therapeutic approaches were consistently associated with a significant reduction in bleeding rates, including ABR and the frequency of hemarthroses [11,12].

These findings were consistent across both replacement and non-replacement therapies, including EHL factor concentrates and emicizumab, confirming their effectiveness in achieving hematologic control. However, this reduction in bleeding did not fully eliminate residual joint risk: persistent musculoskeletal alterations were reported despite reduced hemarthroses in patients receiving EHL treatment [12], while spontaneous joint bleeding was still observed in a subgroup of patients receiving emicizumab prophylaxis [16]. Furthermore, laboratory parameters, including drug levels and global coagulation assays, were not reliable predictors of bleeding risk [16].

### 3.3. Persistence of Musculoskeletal Alterations Despite Hematologic Control

Despite the substantial reduction in bleeding events, multiple included studies consistently reported limited or no improvement in musculoskeletal outcomes, indicating that improved hematologic control does not necessarily translate into complete structural recovery [11,12].

Longitudinal and observational data showed that joint health scores (such as the HJHS), range of motion, and muscle function remained unchanged, particularly in patients with pre-existing arthropathy [12]. This underscores that structural joint damage, once established, follows an irreversible course, even when bleeding is adequately controlled, highlighting the dissociation between hematologic control and musculoskeletal recovery [17].

### 3.4. Evidence of Joint Damage and Subclinical Bleeding

A key finding across several studies was the presence of subclinical joint bleeding and structural damage in the absence of clinically evident hemarthroses [11,13,18].

Imaging-based assessments revealed that a substantial proportion of joints without reported bleeding episodes showed signs of prior hemorrhage, including hemosiderin deposition and synovial hypertrophy [11]. Similarly, studies using MRI and MSK-US identified joint alterations in clinically asymptomatic patients, suggesting ongoing disease activity not captured by traditional clinical measures [13,18].

These findings further support the limitations of relying exclusively on clinical history or ABR for patient assessment [15].

### 3.5. Synovitis as a Key Driver of Joint Damage Progression

Synovitis emerged as a key pathological feature in the included studies, acting both as a consequence of joint bleeding and as a driver of subsequent hemorrhagic episodes [11,19].

Persistent synovial inflammation contributes to a self-perpetuating cycle of bleeding and joint damage, promoting the progression of hemophilic arthropathy. Furthermore, its detection in subclinical stages through imaging techniques reinforces its role as an early marker of disease activity [19,20].

Importantly, synovitis was also detected in asymptomatic joints, further supporting the presence of ongoing subclinical disease activity [21].

### 3.6. Role of Imaging Techniques in Joint Assessment

Imaging techniques, particularly MRI and MSK-US, played a central role in the detection and monitoring of joint involvement [13,15].

MRI remains the reference standard for the comprehensive evaluation of osteochondral damage and deep joint structures. However, its limited accessibility, high cost, and low feasibility for repeated assessments restrict its use in routine clinical practice [13,21].

MSK-US, particularly in the context of point-of-care ultrasound (POCUS), has emerged as a practical and effective tool for joint assessment. It has shown good correlation with MRI findings, particularly in the detection of soft tissue abnormalities such as synovitis and joint effusion [13], and has been associated with the identification of early and subclinical changes [15,21].

However, MSK-US has limitations in the evaluation of deep osteochondral structures, reinforcing its complementary rather than substitutive role relative to MRI [13,21].

### 3.7. Clinical Impact of Ultrasound on Decision-Making

Beyond its diagnostic value, MSK-US has been shown to have a direct impact on clinical decision-making [14].

Prospective real-world data indicate that systematic joint assessment using MSK-US led to changes in therapeutic strategies in a significant proportion of patients. Ultrasound findings had a greater influence than clinical scores alone in guiding therapeutic decisions, including modifications in prophylaxis and rehabilitation strategies [14].

In addition, repeated MSK-US assessments may help improve patients’ awareness of their joint status and promote treatment adherence by providing direct visual feedback [22].

### 3.8. Limitations of Current Assessment Tools

The included studies highlighted important limitations of traditional assessment tools.

Clinical scoring systems and bleeding rates were insufficient to fully capture disease activity, particularly in the context of reduced bleeding associated with modern therapies [13,15,16].

Similarly, laboratory parameters did not reliably reflect joint status nor predict bleeding risk [16].

These limitations support the use of integrated assessment strategies combining clinical evaluation, imaging techniques, and functional measures [13,15,23].

## 4. Advances in Hemophilia Therapies

**Figure 4:**
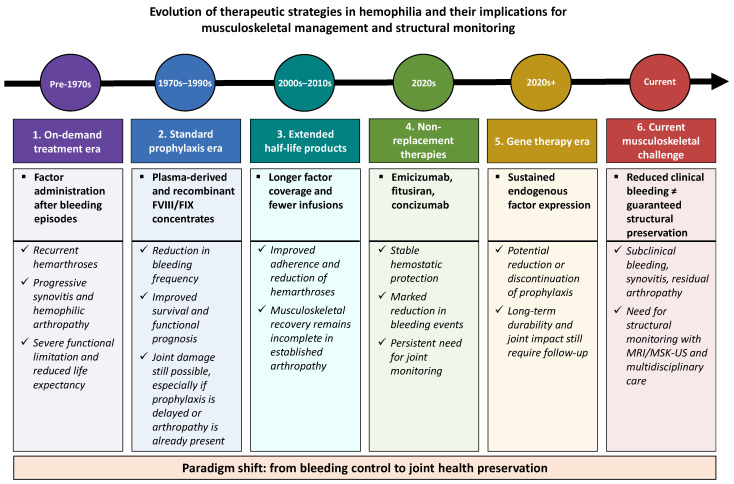
Evolution of therapeutic strategies in hemophilia and their implications for musculoskeletal management and structural monitoring. ABR: annualized bleeding rate; EHL: extended half-life; FVIII/FIX: coagulation factors VIII/IX; MRI: magnetic resonance imaging; MSK-US: musculoskeletal ultrasound.

Hemophilia treatment has undergone a profound transformation over the past two decades, modifying both the natural history of the disease and its clinical and functional management. The most significant change has been the transition from “on-demand” therapy—based on factor administration following a bleeding episode—to regular and personalized prophylaxis aimed at preventing bleeding and preserving joint function, with the additional goal of limiting long-term structural joint damage progression. The evolution of the main therapeutic strategies and their implications for musculoskeletal management and structural monitoring are summarized in Figure 4.

### 4.1. Replacement Therapies: From Plasma-Derived Concentrates to Recombinant Products

Plasma-derived concentrates marked the beginning of effective bleeding control, although they carried a risk of transmission of infectious agents. With the introduction of recombinant concentrates, this risk was virtually eliminated, and dosing became more standardized [1].

EHL products, achieved through molecular modifications (pegylation, fusion with albumin or IgG Fc), allow for less frequent infusions, improved adherence, and longer-lasting protective plasma levels [24,25]. These advances have had a direct impact on reducing the number of hemarthroses and improving patient-reported quality of life, with a consequent reduction in the risk of structural progression detected by imaging techniques in longitudinal studies [25].

### 4.2. Non-Replacement Therapies

Emicizumab, a bispecific antibody that mimics the cofactor function of factor VIII, has represented a major therapeutic paradigm shift. Its stable and prolonged subcutaneous administration has enabled a marked reduction in bleeding frequency, even in patients with inhibitors [26,27], modifying the clinical profile of joint damage observed in routine practice.

Other investigational agents, such as fitusiran (an antithrombin inhibitor) and concizumab (a monoclonal antibody targeting tissue factor pathway inhibitor [TFPI]), modulate hemostatic balance through non-replacement mechanisms and have shown promising results in reducing spontaneous bleeding events [28,29,30,31].

### 4.3. Gene Therapy

Gene therapy represents an unprecedented milestone, with the potential to provide sustained endogenous production of the deficient clotting factor. Clinical trials in hemophilia A and B have demonstrated significant and durable increases in plasma factor levels, reducing or even eliminating the need for prophylaxis [32].

Although these results are promising, uncertainties remain regarding the durability of the effect, immune responses, and patient selection criteria [4]. Nevertheless, its introduction redefines the functional prognosis and calls for a reconsideration of rehabilitation follow-up models, including the need for structural monitoring strategies capable of objectively assessing the real impact of these therapies on joint integrity.

### 4.4. Functional Impact and Quality of Life

The reduction in bleeding episodes translates into a marked decrease in progressive joint damage. However, longitudinal studies show that musculoskeletal function does not fully normalize, particularly in patients with established arthropathy prior to the initiation of prophylaxis [7], highlighting that reduced bleeding does not always result in complete structural recovery.

In addition, a new clinical profile is emerging: young patients with preserved joint structure but with deficits in proprioception and muscle strength, resulting from years of restricted physical activity [7]. This phenomenon underscores the need to integrate adapted therapeutic exercise programs and regular physiotherapy follow-up, supported by objective structural assessment tools capable of detecting subclinical alterations.

The combination of advanced hematologic therapies with structured rehabilitation strategies opens the possibility, for the first time, of achieving comprehensive hemophilia control not only at the hematologic level but also at the functional and preventive levels, provided that this control is complemented by systematic evaluation of joint integrity [33].

## 5. Persistent Musculoskeletal Challenges

**Figure 5:**
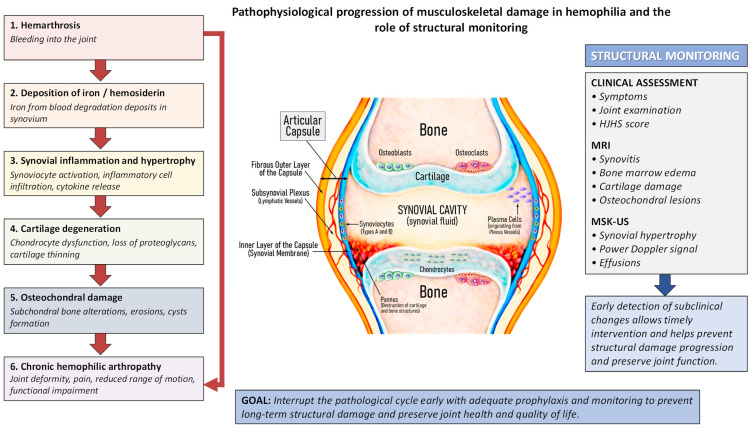
Pathophysiological progression of musculoskeletal damage in hemophilia and the role of structural monitoring. HJHS: Hemophilia Joint Health Score; MRI: magnetic resonance imaging; MSK-US: musculoskeletal ultrasound.

Despite therapeutic advances and the marked reduction in joint bleeding, musculoskeletal problems continue to represent a major source of morbidity and functional limitation in people with hemophilia. The evolving clinical profile has not eliminated pre-existing structural damage or the cumulative effects of decades of recurrent bleeding, which lead to chronic joint alterations. In addition, new challenges are emerging as a result of increased physical activity, longer life expectancy, and residual biomechanical sequelae, highlighting the need for systematic structural assessment to objectively monitor joint evolution over time [33]. The pathophysiological progression of musculoskeletal damage in hemophilia and the role of structural monitoring are summarized in Figure 5.

### 5.1. Pre-Existing Structural Damage and Established Arthropathy

Hemophilic arthropathy represents the final outcome of recurrent joint bleeding and the resulting chronic synovial inflammation [2,20]. Even in patients who are currently well controlled from a hematologic perspective, the sequelae of accumulated damage persist, including stiffness, pain, crepitus, joint axis alteration, and progressive loss of strength and mobility [20,21]. From a structural standpoint, these alterations include persistent synovial hypertrophy, condylar irregularities, and osteochondral changes that may progress even in the absence of clinically evident bleeding [19].

This condition shares similarities with secondary osteoarthritis but also presents specific features, such as vascularized synovial hypertrophy and progressive epiphyseal deformity. These lesions are irreversible and therefore require preventive strategies and continuous physiotherapy to preserve function and delay the need for orthopedic surgery, as well as imaging tools to monitor their stability or progression [20].

### 5.2. Subclinical Bleeding and Hidden Joint Damage

The reduction in clinically visible hemarthroses has highlighted the presence of microbleeding or subclinical bleeding, which may go unnoticed clinically but can sustain synovial inflammation and cartilage degradation [18]. Early identification of these alterations represents one of the main challenges in the modern therapeutic era.

These microscopic lesions, detectable by MSK-US or MRI, are associated with progressive deterioration even in the absence of pain or overt inflammation [15,18]. This finding has changed the approach to physiotherapy follow-up, which is no longer limited to acute episodes but must incorporate sensitive assessment tools and regular monitoring of joint function, integrating structural monitoring systematically into routine clinical practice.

### 5.3. Chronic Pain and Functional Stiffness

Pain in hemophilia is not exclusively of inflammatory or structural origin; neuropathic mechanisms and central sensitization also play a role. The coexistence of chronic synovitis, contractures, and postural alterations leads to compensatory movement patterns that increase mechanical load and the risk of recurrence [19].

Joint stiffness, particularly in the knee and ankle, results in reduced biomechanical efficiency, affecting gait and balance [7]. In turn, this stiffness limits participation in exercise, perpetuating a cycle of inactivity, muscle weakness, and joint risk. Physiotherapy plays a key role in restoring functional movement patterns and preventing pain progression; however, the relationship between symptoms and structural findings requires objective assessment through imaging techniques.

### 5.4. Muscle Weakness and Functional Imbalances

Muscle atrophy secondary to disuse or prolonged immobilization following hemarthrosis is a common finding. Even in patients without recent bleeding episodes, deficits in strength, endurance, and neuromuscular control persist, particularly in the lower limbs [7].

Individualized therapeutic exercise programs have been shown to improve joint function, stability, and quality of life without increasing bleeding risk when appropriately dosed [7]. Patient education is a key component of these programs, promoting self-management and safe participation in physical activity; however, their implementation should be supported by structural assessment to allow appropriate load adjustment and prevent progression of joint damage.

### 5.5. Aging and Emerging Comorbidities

The increased life expectancy of people with hemophilia has led to new clinical scenarios. Aging is associated with cardiovascular, metabolic, and osteoarticular comorbidities that may worsen musculoskeletal symptoms [33].

In addition, the coexistence of joint prostheses, previous surgeries, or longstanding deformities requires adaptation of exercise programs and physiotherapy follow-up. In this context, a preventive approach becomes even more relevant to avoid recurrence and preserve functional independence in later stages of life, requiring structured assessment protocols that include periodic monitoring of joint integrity.

### 5.6. Clinical Implications

The persistence of musculoskeletal sequelae in the era of advanced therapies requires a reconsideration of care objectives. Therapeutic success can no longer be defined solely by the absence of bleeding, but rather by the preservation of joint function, movement capacity, and social participation, which implies incorporating objective structural indicators into routine follow-up.

Physiotherapy and rehabilitation should be systematically integrated into routine patient follow-up, with assessment protocols that include the measurement of joint range of motion, muscle strength, functional capacity, and health-related quality of life [34], complemented by imaging techniques such as MSK-US to achieve a comprehensive evaluation.

## 6. Current Physiotherapy Care Model

Physiotherapy plays a fundamental role in the comprehensive management of hemophilia, both in prevention and functional recovery. The care model has evolved in parallel with hematologic advances, shifting from a reactive approach focused on post-bleeding treatment to a proactive model oriented toward prevention, functional preservation, and patient education, progressively integrating structural assessment tools into routine clinical follow-up.

### 6.1. General Structure of Physiotherapy Care

In specialized hemophilia treatment centers, physiotherapy care is typically organized into two complementary settings:

1. Scheduled or follow-up consultations, where comprehensive functional assessment, therapeutic exercise planning, and self-management education are performed, including structural evaluation when available.
2. Management of acute episodes, focused on recovery following hemarthrosis, orthopedic surgery, or exacerbation of musculoskeletal pain [1,3].

The integration of these two levels of care allows for continuity in patient management, promoting prevention of recurrence and early detection of complications, particularly when supported by objective methods for joint monitoring.

### 6.2. Assessment and Functional Recording

Physiotherapy follow-up should be based on a structured and quantifiable assessment. The most commonly used tools include:

- The clinical HJHS score, which evaluates overall joint status.
- The Haemophilia Early Arthropathy Detection with Ultrasound (HEAD-US) [9], an ultrasound protocol for early detection of synovitis and subclinical damage.
- Pain, mobility, strength, and functional scales adapted to hemophilia [13].

These instruments allow objective monitoring of patient progression and adjustment of exercise programs according to clinical response, while also providing relevant structural information when MSK-US is incorporated into the assessment process.

### 6.3. Therapeutic Exercise and Functional Prevention

Therapeutic exercise is the cornerstone of physiotherapy in hemophilia. Its aim is to maintain joint stability, improve strength and proprioception, and prevent new injuries. Programs should be individually tailored, taking into account the type of hematologic treatment, the level of physical activity, and the presence or absence of pre-existing joint damage [34], ideally supported by prior structural assessment to allow appropriate load adjustment and minimize the risk of joint progression.

Therapeutic exercise programs have been shown to improve joint function, stability, and quality of life without increasing bleeding risk when appropriately dosed [35,36]. In this context, interventions based on progressive strength training have demonstrated improvements in functional capacity and neuromuscular control in patients with hemophilia.

Isometric exercises are safe and effective in early stages or in painful joints, while isotonic and proprioceptive exercises are progressively introduced as joint control improves. Regular practice, combined with educational reinforcement, helps restore patient confidence in movement and break the cycle of inactivity associated with fear of bleeding [37].

### 6.4. Interdisciplinary Coordination

The physiotherapist is an essential member of the multidisciplinary team, working alongside the hematologist, rehabilitation physician, orthopedic surgeon, psychologist, and social worker. Communication among professionals is crucial for clinical decision-making, particularly in situations such as acute bleeding, postoperative rehabilitation, or sports planning [21], as well as for the joint interpretation of structural findings obtained through imaging techniques.

In addition, the physiotherapist’s involvement in patient and caregiver education improves treatment adherence and understanding of joint self-care. In some centers, the role of a “reference physiotherapist” serves as a link between the patient and the rest of the team, ensuring continuity and coherence of care, including coordination of periodic structural monitoring.

### 6.5. Education and Self-Management

Education is a key therapeutic tool. Teaching patients to recognize signs of bleeding, apply joint protection strategies, and perform exercises safely is an essential component of modern physiotherapy [1,7].

Active self-management reduces dependence on the healthcare system and promotes responsible participation in prevention. Education should be adapted to the patient’s age, functional capacity, and level of understanding, involving families and caregivers when appropriate, and facilitating the interpretation of structural findings when imaging techniques are used during follow-up [1,22].

### 6.6. Limitations and Challenges of the Current Model

Although the role of the physiotherapist is well established in specialized hemophilia centers, important limitations persist:

- Lack of standardization in assessment and follow-up protocols.
- Inequities in access to specialized physiotherapy, particularly outside major urban centers.
- Limited availability of MSK-US in physiotherapy settings.
- Insufficient institutional recognition of the physiotherapist’s preventive role.

These limitations highlight the need to move toward a more structured and standardized care model, based on systematic data collection and effective interdisciplinary coordination, with the consistent integration of accessible and reproducible structural monitoring tools.

## 7. Proposed Multidisciplinary Model with Minimum Data Set and Basic Ultrasound

**Figure 6:**
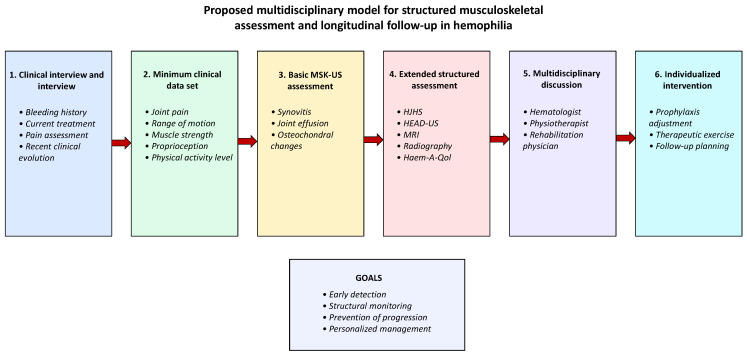
Proposed multidisciplinary model for structured musculoskeletal assessment and longitudinal follow-up in hemophilia. Haem-A-QoL: Haemophilia-Specific Quality of Life Questionnaire; HEAD-US: Haemophilia Early Arthropathy Detection with Ultrasound; HJHS: Hemophilia Joint Health Score; MRI: magnetic resonance imaging; MSK-US: musculoskeletal ultrasound.

The modern management of hemophilia should be inherently multidisciplinary, structured, and prevention-oriented. Although hematologic advances have markedly reduced bleeding episodes, joint function is fully preserved only when continuous rehabilitation follow-up and effective communication among the professionals involved are ensured. In this context, we propose a comprehensive care model centered on the collection of standardized minimum data and the systematic use of basic MSK-US in physiotherapy settings, as a core tool for structural monitoring in routine clinical practice. The proposed multidisciplinary workflow for structured musculoskeletal assessment and longitudinal follow-up is summarized in Figure 6.

### 7.1. Rationale of the Model

The main objective of this model is to enable early detection of musculoskeletal alterations, optimize clinical decision-making, and facilitate comparison of outcomes across centers by incorporating objective structural indicators into longitudinal follow-up. Clinical experience shows that direct observation and physical examination alone are not always sufficient to identify synovitis or subclinical bleeding [15].

The integration of MSK-US allows objective visualization of cartilage integrity, the presence of joint effusion or synovial hypertrophy, and assessment of the progression of hemophilic arthropathy without the need for more invasive or costly imaging techniques [34], positioning it as an accessible and repeatable tool for periodic structural monitoring. The proposed model is conceptually aligned with current international recommendations emphasizing multidisciplinary care, early prevention, functional preservation, and structured musculoskeletal assessment in hemophilia [1,7].

### 7.2. Structure of the Multidisciplinary Visit

The proposed visit combines clinical, physiotherapy, and MSK-US assessment within a single session. This format facilitates communication among professionals and avoids duplication of care, allowing immediate integration of structural findings into clinical decision-making.

During the visit, the physiotherapist or specialized rehabilitation physician performs:

- Structured clinical interview, including bleeding history, current treatment, and recent clinical evolution.
- Recording of minimum clinical and functional data for structured follow-up of the main target joints, which may be complemented with functional scales such as HJHS or quality-of-life questionnaires (Haemophilia-Specific Quality of Life Questionnaire [Haem-A-QoL], EuroQol-5 Dimensions [EQ-5D]) when considered necessary.
- Basic MSK-US assessment focused on the main target joints (knees, ankles, and elbows), aimed at detecting synovitis, joint effusion, and early osteochondral changes through a structured examination based on targeted high-prevalence scans, scalable to more comprehensive ultrasound tools such as HEAD-US as well as to other imaging techniques such as MRI or radiography when clinically required.
- Immediate discussion with the hematology team, to adjust prophylaxis or plan complementary interventions based on the findings, promoting a care model guided by objective structural evidence.

This organization enables integrated, patient-centered decision-making, reducing response time to acute issues or alterations detected during follow-up.

### 7.3. Content of the Minimum Data Set

The proposed model includes the development of a unified registry of clinical and functional data focused on the main target joints (knees, ankles, and elbows), incorporating the following key items:

- Presence of joint pain (Visual Analog Scale [VAS]).
- Active and passive range of motion.
- Muscle strength (manual testing or dynamometry).
- Presence of synovitis, joint effusion, or basic structural alterations on MSK-US.
- Level of physical activity and therapeutic exercise performed.

Recording these parameters at each visit allows longitudinal evaluation of patient progression and enables comparisons across centers, contributing to research and improving quality of care, with particular relevance of structural data as potential markers of joint progression.

### 7.4. Role of the Physiotherapist in Model Implementation

The physiotherapist plays a key role both in basic MSK-US assessment and in the functional interpretation of findings. The combination of expertise in biomechanics and joint pathophysiology allows correlation between imaging findings and clinical symptoms, facilitating the planning of individualized preventive strategies by integrating structure and function within a single clinical approach.

The progressive training of physiotherapists in MSK-US—already implemented in several European and Latin American centers—improves system efficiency and reduces dependence on external diagnostic tests. In addition, direct communication with the hematologist facilitates treatment adjustments and helps prevent lesion chronicity, reinforcing the role of ultrasound as a dynamic follow-up tool [7,15].

### 7.5. Benefits of the Proposed Model

The potential benefits of this proposal include:

- Early detection of subclinical lesions and prevention of irreversible damage.
- Standardization of assessment protocols across centers.
- Optimization of clinical time through the integration of professionals within a single visit.
- Improvement in patient education and adherence through the provision of consistent and coordinated information.
- Generation of comparable data useful for research and clinical audits, particularly regarding the evolution of structural joint changes.

### 7.6. Challenges and Requirements for Implementation

The implementation of this model requires overcoming several challenges:

- Specific training of physiotherapists in MSK-US.
- Adequate technical and human resources in treatment centers.
- Institutional recognition of the physiotherapist’s functional diagnostic role.
- Development of collaborative national and international networks for data sharing and experience exchange.

The standardization of musculoskeletal assessment in hemophilia is a key step toward consolidating the new therapeutic paradigm, in which systematic structural monitoring complements hematologic control. This model not only improves clinical care but also contributes to the development of multicenter research and the establishment of evidence-based clinical practice guidelines.

### 7.7. Technical Considerations for MSK-US in Hemophilia

To ensure consistency and reproducibility of the proposed model, ultrasound assessment should be based on standardized technical principles. The most frequently explored target joints in hemophilia are the knees, ankles, and elbows, due to their high prevalence of involvement [1].

Examination should be performed using high-frequency linear transducers, with systematic evaluation in longitudinal and transverse planes, including assessment of: (1) joint effusion; (2) synovial hypertrophy and degree of vascularization when Doppler is available; (3) integrity of the hyaline cartilage; and (4) osteochondral irregularities or subchondral structural changes.

Ultrasound also allows dynamic and bilateral comparative assessment, facilitating early detection of subtle alterations even in the absence of clinical symptoms. In routine clinical practice, simplified structured ultrasound approaches based on a reduced number of predefined scans targeting high lesion-prevalence recesses, integrated with structured clinical data, may improve feasibility and facilitate longitudinal follow-up [9,38,39,40]. Whenever possible, ultrasound assessment may be complemented with standardized acquisition protocols and scanning approaches, such as HEAD-US [9], to improve reproducibility and facilitate longitudinal comparison between evaluations in clinical practice; however, the proposed model does not rely exclusively on a scoring system, but rather on the structured integration of clinical and structural findings within longitudinal follow-up.

Although ultrasound is particularly useful for identifying joint effusion, synovial hypertrophy, and superficial osteochondral changes, its ability to characterize certain deep structural alterations may be limited depending on the joint and the available acoustic window. In cases of discordance between clinical findings and ultrasound results, or when complex involvement is suspected, MRI may be considered as a complementary technique for more detailed structural characterization [13,20].

## 8. General Discussion

The findings of this structured narrative review, conducted following PRISMA methodological principles (although not designed as a systematic review), confirm a relevant shift in the clinical paradigm of hemophilia. Despite recent therapeutic advances, particularly with the use of EHL factor concentrates and non-replacement agents such as emicizumab, the available evidence consistently shows that improved hematologic control does not necessarily translate into complete preservation of joint structural integrity.

In this regard, multiple studies included in this review demonstrate a dissociation between the reduction in bleeding episodes and the progression of hemophilic arthropathy, particularly in patients with pre-existing joint damage [11,12]. Although a significant reduction in ABR is observed, joint health parameters such as HJHS, range of motion, and muscle function show limited or even no improvement in the medium and long term [12].

This finding is further supported by real-world data, where the use of EHL factor concentrates is associated with reductions in bleeding rates without clear population-level differences in joint health or quality-of-life measures [41], potentially suggesting persistence of the dissociation between hematologic control and joint status. However, other studies have reported improvements in health-related quality of life associated with modern prophylactic strategies, particularly regarding physical activity and psychosocial well-being [5], suggesting that these outcomes may vary depending on patient characteristics, baseline joint status, and assessment methodology.

This phenomenon may be explained, at least in part, by the progressive and irreversible nature of osteoarticular damage once established. The findings of this review suggest that current therapies are highly effective in preventing new clinical bleeding episodes, but not in reversing existing structural damage [20].

In addition, a key aspect identified is the presence of subclinical joint damage in patients without apparent bleeding episodes. Several studies show that imaging techniques such as MRI and, particularly, MSK-US can detect synovitis, synovial hypertrophy, and osteochondral changes in apparently asymptomatic joints [18].

This phenomenon is further supported by studies demonstrating that, even under effective emicizumab prophylaxis, microhemorrhages and underlying inflammatory processes may persist, contributing to progressive osteoarticular deterioration [42].

These findings support the hypothesis that recurrent microbleeding or persistent inflammatory processes may exist without being detected by traditional clinical tools. In this context, several studies agree that classical indicators such as ABR or even certain hematologic parameters are not reliable predictors of joint status or the risk of disease progression [16].

In contrast, structural assessment through imaging techniques emerges as a key element in patient follow-up. In particular, the detection of synovitis by ultrasound has been associated with an increased risk of joint bleeding and progression of arthropathy, even in patients under optimized prophylaxis [19].

In this context, MSK-US acquires a strategic role within the new clinical management model. Its accessibility, dynamic nature, and ability to be repeatedly applied make it a particularly useful tool in routine clinical practice, enabling early detection of structural alterations and individualized treatment adaptation [15].

From a clinical perspective, the implications of structural monitoring may vary according to the therapeutic strategy used. In patients treated with standard or EHL factor concentrates, exercise planning and musculoskeletal follow-up may remain closely linked to pharmacokinetic coverage and timing of administration. In contrast, non-replacement therapies such as emicizumab provide a more stable hemostatic profile, modifying the traditional relationship between treatment timing and physical activity, but not eliminating the need for functional and structural surveillance [26,27]. In the context of gene therapy, the possibility of sustained endogenous factor expression introduces a new scenario; however, uncertainties regarding durability and interindividual variability reinforce the need for objective longitudinal monitoring of joint integrity [32].

Furthermore, some studies suggest that direct visualization of joint status through ultrasound may have a positive impact on treatment adherence, facilitating patient understanding of structural damage and reinforcing engagement in follow-up [22].

However, it is important to consider that ultrasound has limitations in the assessment of deep osteochondral structures, where MRI remains the reference standard. Therefore, both techniques should be understood as complementary within a multimodal assessment approach [13].

Another relevant aspect emerging from this review is the changing clinical profile of patients with hemophilia. Increased life expectancy has led to an aging population with a higher burden of comorbidities, including cardiovascular, metabolic, and degenerative conditions, which may influence musculoskeletal evolution and response to treatment [21].

In this context, recent studies also show that combining hematologic therapies with interventions such as structured therapeutic exercise can improve musculoskeletal parameters and quality of life, reinforcing the active role of rehabilitation in the current era [43].

This new clinical scenario reinforces the need for more complex and integrated care models, in which structural monitoring and physiotherapy intervention play a central role. The combination of clinical assessment, functional tools, and imaging is presented as the most appropriate strategy to comprehensively address joint health in these patients.

Overall, the findings of this review support the need to evolve from a model focused exclusively on bleeding control toward a broader approach that integrates structural and functional joint assessment as key elements in clinical decision-making.

## 9. Limitations

This structured narrative review presents several limitations that should be considered when interpreting the findings. Although the review was conducted following PRISMA 2020-informed methodological principles, no formal risk of bias assessment of the included studies was performed, which limits the ability to establish robust causal relationships between the analyzed variables. In addition, the search was restricted to two main databases, a defined time frame, and title/abstract-based search fields, which may have limited the inclusion of potentially relevant literature and reduced the overall comprehensiveness of the evidence compared with broader systematic search strategies. However, this approach was deliberately adopted to prioritize clinically focused studies directly related to musculoskeletal involvement and structural monitoring in hemophilia.

Furthermore, the methodological heterogeneity of the included studies, in terms of design, population, and assessment tools, complicates direct comparison of results and limits the possibility of drawing quantitative conclusions or performing robust statistical syntheses, such as meta-analyses.

In this context, although a systematic and reproducible selection process was applied, the final inclusion of studies was also influenced by criteria of clinical relevance, inherent to the structured narrative design of the review, which may introduce a certain degree of subjectivity in the interpretation and hierarchical weighting of the available evidence. Therefore, explicit differentiation between structured musculoskeletal outcomes directly measured/evaluated and broader thematic musculoskeletal concepts was incorporated throughout the methodological approach, figures, and evidence synthesis in order to preserve transparency and avoid overinterpretation of evidence levels.

Additionally, it should be considered that the available evidence is largely based on observational studies and real-world clinical practice. While this enhances the external validity of the findings, it may also be associated with limitations in controlling confounding variables and in the standardization of outcome measures.

Nevertheless, despite these limitations, the structured approach and the consistency of the findings allow the identification of clinically relevant patterns and provide a solid basis for understanding the musculoskeletal impact of advanced therapies in hemophilia.

## 10. Conclusions

Over the past few decades, hemophilia has evolved from a highly disabling disease into a manageable condition that allows for an active life with functional expectations comparable to those of the general population. However, this therapeutic progress does not automatically translate into complete recovery of the musculoskeletal system or elimination of joint risk, highlighting the need for systematic structural monitoring in the modern therapeutic era.

New hematologic treatments—including EHL factor concentrates, bispecific antibodies, and gene therapy—have significantly reduced bleeding frequency. Nevertheless, multiple studies indicate that this improvement does not result in a meaningful recovery of joint health parameters, nor in the complete resolution of accumulated sequelae or subclinical microbleeding. Therefore, physiotherapy and rehabilitation remain central and irreplaceable components of comprehensive care, supported by objective tools that allow correlation between clinical evolution and structural joint integrity.

Preserving joint function and quality of life requires a comprehensive approach that integrates prevention, education, and early intervention. The incorporation of MSK-US into physiotherapy practice represents a major advancement in this regard, providing an objective assessment tool capable of detecting subtle alterations and enabling personalized intervention strategies.

The proposed multidisciplinary model, based on the collection of minimum data and fluid communication among hematologists, physiotherapists, rehabilitation physicians, and orthopedic specialists, constitutes an effective strategy to consolidate therapeutic gains and prevent progression toward chronic arthropathy by systematically integrating structural monitoring into routine clinical follow-up. Only through coordinated clinical efforts and systematic data collection can equitable, high-quality care be ensured for all individuals with hemophilia, regardless of healthcare setting.

Ultimately, the shift in the hematologic paradigm requires a parallel transformation in the rehabilitation paradigm: from a reactive approach to one that is preventive, continuous, and function-oriented. In this context, the future of hemophilia care will depend not only on pharmacological innovations, but also on the ability of clinical teams to integrate structural monitoring strategies into routine practice. The systematic incorporation of tools such as MSK-US should therefore be considered not as an adjunct, but as an essential component to ensure that therapeutic advances translate into sustained preservation of joint integrity and long-term functional outcomes.

## 11. Future Perspectives

In light of the limitations identified in clinical monitoring and early detection of joint damage, the future of rehabilitation in hemophilia is moving toward increasingly personalized, digitalized, and evidence-based models, in which structural monitoring will play a central role within comprehensive clinical follow-up.

The consolidation of ultrasound as a routine tool, together with the expansion of telemonitoring technologies, will open new possibilities for remote follow-up and continuity of care, enabling periodic and accessible assessment of joint integrity [15].

Key areas for future development include:

- Implementation of telerehabilitation programs to assess and supervise therapeutic exercise at home, ensuring safety and adherence, complemented by structural monitoring systems when feasible [44].
- Integration of artificial intelligence and data analytics into clinical registries to identify patterns of progression and predict individual joint risk, including structural variables obtained through MSK-US [45].
- Multicenter validation of the minimum data set model, involving national and international networks of specialized physiotherapists and incorporating standardized criteria for structural assessment.
- Advanced training in MSK-US for physiotherapists and rehabilitation physicians, ensuring consistency and diagnostic quality, and promoting reproducible standards for image acquisition and interpretation.
- Genuine interdisciplinary collaboration among clinical teams, researchers, and patient organizations to generate practical evidence applicable across different healthcare settings, aimed at consolidating structural monitoring as a core component of the care model.

These strategies will consolidate physiotherapy as a fundamental pillar within the evolving therapeutic landscape, ensuring that hematologic advances translate into sustained functional benefits through objective and systematic structural assessment. The future of hemophilia care will be shaped not only by biotechnology, but also by movement, prevention, and shared clinical knowledge, with musculoskeletal imaging integrated as a strategic tool for longitudinal follow-up.

## Figures and Tables

**Table 1:** Characteristics of the included studies.

Author, Year	Study Design	Population	Intervention/Treatment	Variables Assessed	Main Findings
Shima et al., 2025 [11]	Multicenter clinical study	Pediatric, hemophilia A	Emicizumab	ABR, HJHS, MRI	Reduced bleeding; persistent joint damage
Youssry et al., 2024 [13]	Cross-sectional study	Hemophilia in a low-resource setting	Different prophylaxis regimens	Clinical assessment + MSK-US	MSK-US underestimates vs. MRI; subclinical damage detected
Cuesta-Barriuso et al., 2022 [12]	Prospective cohort study	Severe hemophilia A	EHL factors	HJHS, ROM, muscle function	Improved bleeding; no structural recovery
Arcudi et al., 2024 [16]	Observational study	Hemophilia A	Emicizumab	ABR, laboratory parameters	Residual bleeding; poor lab prediction
Théron et al., 2024 [17]	Review	Hemophilia	Mesenchymal stromal cell-based therapies	Pathophysiological mechanisms	Potential in advanced damage; limited regeneration
Rodríguez-Merchán, 2023a [18]	Review	Hemophilia	Early diagnosis	Imaging	Subclinical damage relevant
Rodríguez-Merchán, 2023b [19]	Review	Hemophilia	Synovitis	Imaging + clinical	Synovitis drives progression
Minno MNDD et al., 2023 [15]	Review	Hemophilia	POCUS	Structural assessment	MSK-US useful; ABR limitations
Gualtierotti et al., 2021 [20]	Review	Hemophilia	Global approach	Pathophysiology	Structural progression persists
Bakeer et al., 2024 [21]	Review	Adults with hemophilia	Comprehensive management	Clinical + comorbidities	Aging impact; persistent arthropathy
Di Minno A et al., 2019 [22]	Conceptual/clinical study	Hemophilia	Repeated MSK-US	Adherence, imaging	Improved adherence via imaging
Rodríguez-Merchán, 2021 [23]	Review	Hemophilia in developing countries	Educational approach	Clinical assessment	Limited structural assessment
Drillaud et al., 2025 [14]	Prospective observational study	Hemophilia A	Systematic assessment (MSK-US + clinical)	Treatment decisions	MSK-US influences decisions

ABR: annualized bleeding rate; HJHS: Hemophilia Joint Health Score; MRI: magnetic resonance imaging; MSK-US: musculoskeletal ultrasound; ROM: range of motion; EHL: extended half-life products; POCUS: point-of-care ultrasound.

**Table 2:** Summary of original clinical study populations.

Study	Sample Size
Shima et al., 2025 [11]	30
Youssry et al., 2024 [13]	50
Cuesta-Barriuso et al., 2022 [12]	46
Arcudi et al., 2024 [16]	40
Drillaud et al., 2025 [14]	86
**Total**	**≈252**

Only studies with directly quantifiable original patient cohorts were included; review and conceptual articles were not considered suitable for cumulative population estimation.

## Data Availability

No new data were created or analyzed in this study.

## Supplementary Materials

The following supporting information can be downloaded at: https://www.mdpi.com/article/10.3390/diagnostics16111683/s1, PRISMA 2020 checklist.
